# *Ornithobacterium rhinotracheale*: MALDI-TOF MS and Whole Genome Sequencing Confirm That Serotypes K, L and M Deviate from Well-Known Reference Strains and Numerous Field Isolates

**DOI:** 10.3390/microorganisms9051006

**Published:** 2021-05-07

**Authors:** Merima Alispahic, Lukas Endler, Michael Hess, Claudia Hess

**Affiliations:** 1 Clinic for Poultry and Fish Medicine, Department for Farm Animals and Veterinary Public Health, University of Veterinary Medicine, 1210 Vienna, Austria; michael.hess@vetmeduni.ac.at (M.H.); claudia.hess@vetmeduni.ac.at (C.H.); 2 Platform Bioinformatics and Biostatistics, Department of Biomedical Sciences, University of Veterinary Medicine, 1210 Vienna, Austria; lukasendler@gmail.com

**Keywords:** *Ornithobacterium rhinotracheale* ORT, turkeys, MALDI-TOF MS, whole genome sequencing WGS, diagnosis, microorganism identification

## Abstract

*Ornithobacterium rhinotracheale* is one of the most important bacterial agents of respiratory diseases in poultry. For correct identification and characterization of this fastidious bacterium, reliable diagnostic tools are essential. Still, phenotypic tests are used to identify *O. rhinotracheale* and serotyping is the most common method for characterization, despite known drawbacks and disadvantages such as divergent results, cross-reactivity between strains, or the non-typeability of strains. The intention of the present study was to evaluate MALDI-TOF MS and whole genome sequencing for the identification and characterization of *O. rhinotracheale*. For this purpose, a selection of 59 well-defined reference strains and 47 field strains derived from outbreaks on Austrian turkey farms were investigated by MALDI-TOF MS. The field strains originated from different geographical areas in Austria with some of the isolates derived from multiple outbreaks on farms within a year, or recurrent outbreaks over several years. MALDI-TOF MS proved a suitable method for identification of *O. rhinotracheale* to genus or species level except for 3 strains representing serotypes M, K and F. Phylogenetic analysis showed that most strains grouped within one cluster even though they were comprised of different serotypes, while serotypes F, K, and M clearly formed a different cluster. All field isolates from turkey farms clustered together, independent of the origin of the isolates, e.g., geographical area, multiple outbreaks within a year or recurrent outbreaks over several years. Whole genome sequencing of serotype M, K and F strains confirmed the extraordinary status and deviation from known fully-sequenced strains due to a lack of sequence similarity. This was further confirmed by alignments of single genes (16S-RNA and rpoB) and multilocus sequence typing although the demarcation was less obvious. Altogether, the results indicate that these three serotypes belong to a different species than *O. rhinotracheale*, and might even be members of multiple new species.

## 1. Introduction

*Ornithobacterium rhinotracheale* is a gram-negative rod-shaped bacterium firstly described in 1994 [1], and it was the only species in its genus until 2019 when Salter et al. [2] proposed a new species, *Ornithobacterium* hominis, isolated from humans. *O. rhinotracheale* is an economically important bacterial pathogen of turkeys and chickens worldwide [3], and it is ranked among the most important bacterial agents of poultry respiratory diseases [4]. Apart from commercial poultry, infections with *O. rhinotracheale* are also reported from many other bird species [5,6,7,8,9,10,11,12,13]. In this context, wild birds are regarded as a potential source of infection for commercial poultry flocks [14].

The clinical picture is, besides respiratory symptoms, mainly associated with growth retardation, reduction of egg production, increased mortality and higher condemnation rates [3]. Gross pathological lesions are characterized by pneumonia, pleuritis, and airsacculitis.

Infections with *O. rhinotracheale* are primarily diagnosed by bacterial isolation followed by identification and/or detection of antibodies [3]. Since its first detection, a variety of typing methods have been used to gain basic knowledge about the population structure of *O. rhinotracheale*. Differentiation of strains based on phenotypic methods often resulted in heterogeneous outcomes or non-typeable strains [6,15]. Serological characterization revealed at least 18 different serotypes (A–R). So far, the most frequently isolated strains belong to serotype A in chickens and serotypes A, B, D, and E in turkeys. Serotypes F, K, and M are only occasionally isolated from chickens and turkeys [16,17]. Serotyping, however, is impeded by inconsistent results or cross-reactivity between strains limiting its applicability [11,18]. To overcome these disadvantages, a wide range of molecular techniques were implemented over the last years. This contributed to a better understanding of the phylogenetic relationship and indicated the existence of a greater genetic variability [11,12,13,19,20] particularly between strains from different hosts [6,10].

In the last decade, matrix-assisted laser desorption ionization–time of flight mass spectrometry (MALDI-TOF MS) has become popular in routine diagnostic laboratories due to its fast and reliable direct identification of microorganisms. Thus, progressively replacing all the biochemical (e.g., API gallery) and phenotypic tests [21,22] for species characterization.

In the present investigation, we report for the first time the application of MALDI-TOF MS to analyze and differentiate numerous *O. rhinotracheale* strains. The study is based on a selection of well-defined reference strains to see if proteomics has the power to identify and distinguish *O. rhinotracheale* serotypes. Furthermore, selected field strains from *O. rhinotracheale* outbreaks in turkey flocks originating from (a) different geographical areas in Austria, (b) multiple outbreaks within a year on the same farm, (c) recurrent outbreaks over several years noticed on the same farm were included in the study to investigate the relationship of strains. Additionally, whole genome sequencing (WGS) was used to characterize serotype F, K and M strains in order to gain more insights into the genetic diversity of *O. rhinotracheale*.

## 2. Materials and Methods

### 2.1. Bacterial Isolates

In total, 59 well-defined *O. rhinotracheale* reference strains were obtained from LMG (Laboratorium voor Microbiologie, Universiteit Ghent, Ghent, Belgium). Information regarding the serotype was provided for 19 strains comprising 15 (A–O) different serotypes (Table 1). Additionally, 47 field strains isolated from 2002 up to 2016 from *O. rhinotracheale* outbreaks on 31 turkey farms were included. Isolates from multiple outbreaks within a year were investigated from farms 4, 8, 9, 19 and 24. Additionally, farms 1, 2, 10, 18, 21, 24 and 27 faced several recurrent outbreaks over years, of which isolates were included in the investigation.

All strains were stored at −80 °C by adding 2 mL of 40% glycerol/10 mL Brain Heart Infusion Broth (Oxoid, ThermoFisher Scientific, Vienna, Austria). Before investigation, the strains were thawed and cultivated on blood agar (Columbia agar containing 5% sheep blood, BioMeriéux, Vienna, Austria), at 37 °C for 24 h under microaerophilic conditions (Genbox microaer, BioMèrieux, Vienna, Austria).

### 2.2. MALDI-TOF MS

Sample preparation for MALDI-TOF MS was performed as previously described in detail [21]. Bacterial acid-soluble proteins were extracted using formic acid (70%) and acetonitrile according to the standard protocol from Bruker (Daltonics GmbH, Bremen, Germany). One microliter (1µL) of each bacterial extract was spotted onto the MALDI target plate and air-dried. Afterwards, 1 μL matrix solution (alpha-cyano-4-hydroxycinnamic acid in 50% acetonitrile/2.5% trifluoroacetic acid) was spotted on top of each dried sample and left to dry again. The parameter settings for the Microflex LT instrument were as follows: IS1, 20.08 kV; IS2, 16.60 kV; lens, 7.00 kV; detector gain, 2974 V. Two hundred and forty laser shots in 40 shot steps (in the linear, positive ion mode with a 60 Hz nitrogen laser from different positions of the target spot) were summarized automatically with the AutoXecute (MBT AutoX method) acquisition control software (Flex control 4; Bruker Daltonics). For automated data analysis, the raw spectra for unknown bacteria were processed using MALDI Biotyper software (Bruker Daltonics GmbH, Bremen, Germany) with the default settings. The software performs smoothing, normalization, baseline subtraction, and peak picking, thereby creating a list of the most significant peaks (*m/z* values) of the spectrum. For species identification, the MALDI Biotyper output is a log (score) in the range of 0 to 3.0, computed by comparison of the peak list for an unknown isolate with the reference Main Spectra (MSP) in the reference database. A MALDI score between 1.7 and 2.0 represents genus identification, while a MALDI score ≥2.0 represents identification at species level. Anything less than 1.7 was rated as non-identifiable by the software. For database construction or MSP creation, from each bacterial strain acid-soluble proteins were spotted on the MALDI target plate eight times and each spot was measured three times ending with 24 spectra for each strain. FlexAnalysis (v. 4) software (Bruker Daltonics GmbH, Germany) was used for visual inspection of the mass spectra and atypical spectra were excluded from further analysis (e.g., flat line spectra, spectra containing high matrix background signal). For creating a new database entry or an MSP, 20 to 24 mass spectra were processed with the software functionality and standard settings. The spectral peak lists for a particular strain were transferred into MSP containing information on average peak masses, average peak intensities and peak frequencies. Similar MSP result in a high matching score value. Each MSP is compared with all MSP of the analyzed set. The list of score values is used to calculate normalized distance values between the analyzed species, resulting in a matrix of matching scores. The visualization of the respective relationship between the MSP is displayed in a dendrogram using the following settings of the MALDI Biotyper 4.1 software: distance measure was set to correlation, linkage to average and score threshold value for a single organism at 700. Based on Sauer et al. [22], clusters of strains with distance levels <500 were classified as species. For strain relationship visualization, a dendrogram was formed based on MSP.

### 2.3. Sequencing, Genome Assembly and Annotation

Genomic DNA of strains was isolated using QIAGEN DNeasy kits. Libraries were sequenced in a multiplexed 300 bp paired-end run on an Illumina MiSeq (Vienna BioCenter Core Facilities GmbH, Vienna, Austria). For assembly, adapter sequences and low-quality nucleotides were first removed from the 3′ ends using BBduk (BBTools software suite v.37.77 [23], parameters: minlen = 40 qtrim = r trimq = 15 ktrim = r mink = 10 tbo) with the default Illumina and Nextera adapter file, a trimming quality of 15 and a minimal residual length of 40. As the average fragment size for all libraries was small (~250 bp) compared to the read length (300 bp), we also used the trim-by-overlap option (tbo), to remove adaptor and linker sequences on both ends. For estimating genome sizes, sequencing depths and repetitive regions, kmers of length 31 were counted using the kmercountexact.sh script (also BBTools).

As the library fragment sizes turned out to be too small for assembling the ribosomal RNA operons, we used riboSeed (v. 0.4.65) [24] with the sequence of *O. rhinotracheale* DSM 15997 (Refseq: NC_018016.1) as a reference for the rRNA operons. Shortly, Riboseed uses Barrnap [25] to scan for rRNA operons in a closely related reference genome and then—using these rRNA operon regions—extract matching reads and assembles them into pseudo contigs using SPAdes (v. 3.9.0) [26] in an iterative process. The resulting pseudo contigs were then used as trusted contigs in a final assembly round using all reads in SPAdes with a coverage cutoff of 30 (options: -k 21,31,55,77,99,127 --cov-cutoff 30).

To assess the completeness and quality of the genomes, the contigs of all genomes were screened for selected single-copy orthologues using BUSCO (v3) [27,28] with the *bacteroidetes_odb9* dataset. For comparability, we also screened the three already published *O. rhinotracheale* genomes (Additional file S1. Assembly Statistics).

The final contigs were then submitted to GenBank under the BioProject PRJNA501809 with sample IDs SAMN10346233, SAMN10346254, and SAMN10346265, and annotated using the NCBI Prokaryotic Genome Annotation Pipeline (v. 4.6) [29]. The assemblies are available at the NCBI under the GenBank IDs GCA_009659665.1, GCA_009659645.1 and GCA_009659705.1.

The genomes were scanned for antibiotic resistance genes using the online and the command-line version of the RGI tool (v. 5.0) for the CARD database (v. 3.2) [30,31] (Additional file S2. Antibiotic Resistance CARD).

To examine the relatedness of the newly sequenced bacteria to characterized strains of *O. rhinotracheale* and Cand. *O. hominis* (*O. sp. OH-22767* & *OH-2280*), first the average nucleotide identity (ANI) between pairs of sequences was calculated using the OrthoANI tool v. 0.93.1 [32].

The UBCG (up-to-date bacterial core gene) tool v. 3.0 [33] was used to further examine phylogenetic relationships between the fully sequenced *O. rhinotracheale* strains and Cand. *O. hominis* using the genome of *Riemerella anatipestifer* (DSM 15868, Genbank: NC_017045.1) as an out-group. UBCG consists of a predefined set of 92 core genes, which have been found to occur in single copy in an extensive collection of fully sequenced bacterial genomes. The UBCG tool matches and extracts sequences homologous to these 92 core genes, aligns them individually and constructs both single gene and concatenated phylogenetic trees. For construction of the various trees RAxML v. 8.2.12 [34] with a general time-reversible model with gamma-distributed rate variation (GTRGAMMA) and 1000 bootstraps was used. From the individual gene trees, a gene support index (gsi) was calculated, indicating how many of the gene trees support each split of the final concatenated tree. The gene encoding *tig* (Trigger factor) was not identified in any genome, so in the end, only 91 core genes were used.

For a more defined grained look, the sequences of the 16S rRNA and the beta subunit of RNA polymerase (*rpoB*) from the fully sequenced *O. rhinotracheale* genomes were aligned to 16S rRNA sequences of various *O. rhinotracheale* strains using MAFFT (v. 7.429) [35] with iterative refinement and local paired alignment information (--localpair --maxiterate 1000). All regions containing gaps were removed from the alignment using trimAL [36]. The maximum likelihood tree was calculated using RAxML v. 8.2.12 [34] with a general time-reversible model with gamma-distributed rate variation (GTRGAMMA) and 1000 bootstraps using Cand. *O. hominis* (*O. sp. OH-22767* & *OH-2280*) as an out-group.

To compare the newly sequenced genomes to already known multilocus sequence types (MLST) [37], we tried to assess MLSTs using a whole genome MLST typer (v2.0.1) [38], but could not obtain MLST for all regions, as some are too divergent (Additional file S3. MLST typing). Therefore, the *O. rhinotracheale* MLST regions were downloaded from pubmlst [10,39] and the corresponding regions in the newly sequenced strains identified using blast v. 2.9.0 [40] and extracted. These regions were then concatenated and aligned to all known MLST region sequences using MAFFT v. 7.390 [35] with a maximum of 1000 iterations. Again, the maximum likelihood tree was calculated using RAxML v. 8.2.12 [34] with a general time-reversible model with gamma-distributed rate variation (GTRGAMMA) and 1000 bootstraps.

Graphics were created using R (R core team, 2019), pheatmaps [41]. Phylogenetic trees were plotted using ggtree [42].

## 3. Results and Discussion

MALDI-TOF MS was previously demonstrated as a valuable tool to differentiate species of poultry pathogens and investigate their phylogenetic relationship, exemplarily shown for species of the genera *Avibacterium* and *Gallibacterium* [21,43]. Also, in the presented study a reproducible signal pattern was obtained from all *O. rhinotracheale* strains investigated. Signal patterns obtained were compared with data in the Bruker Biotyper reference database version 4 consisting of more than 7000 microorganisms. This resulted in an identification of all strains at the species level, except for the reference strain representing serotype M (LMG 19032), which was identified only to the genus level (MALDI score 1.90), and reference strains of serotypes K and F (LMG 18,856 and 18861), which were not identifiable (MALDI score 1.69, 1.54 respectively) (Table 1).

**Figure 1:**
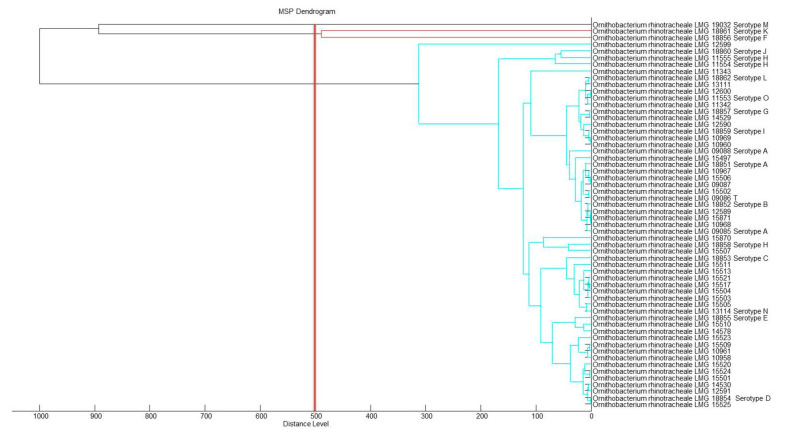
MSP dendrogram of 59 reference strains based on MALDI-TOF MS. Serotypes A, B, C, D, E, G, H, I, J, L, N are concentrated in one cluster. Serotypes F, M and K group into a separate cluster.

**Figure 2:**
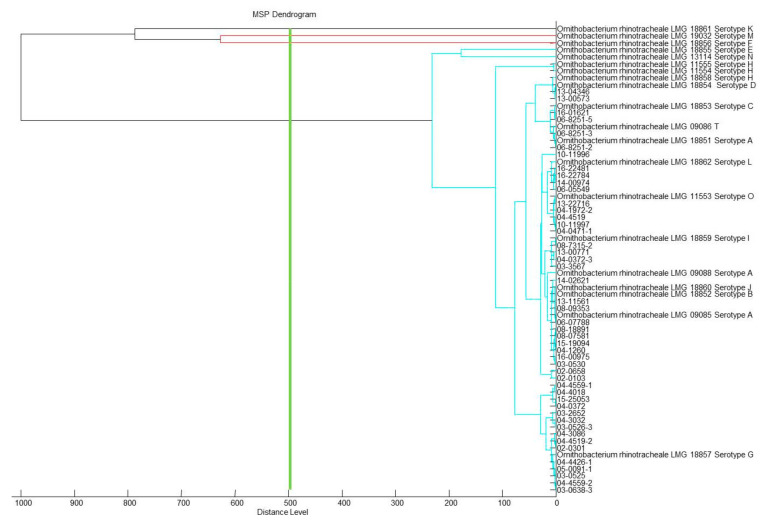
MSP dendrogram based on MALDI-TOF MS of 47 field strains isolated from different geographical areas in Austria and different years.

All 47 field isolates were identified to species level by MALDI-TOF MS (Table 2). The organism’s best MALDI score (Table 1 and Table 2) confirmed the relationship results observed in the MSP dendrogram (Figure 1 and Figure 2). MSP dendrogram (Figure 1) revealed that the 56 reference strains including 12 different serotypes (A, B, C, D, E, G, H, I, J, L, N and O) form one cluster by MALDI. Figure 2 shows that all isolates from multiple or recurrent infections from farms 1, 2, 4, 8, 9, 10, 18, 19, 21, 24 and 27 belong to the same cluster independent from their geographical origin or their year of isolation. Also, no differences between such isolates were detected. These findings agree with previous phylogenetic studies supporting the assumption that a local *O. rhinotracheale* population is predominantly clonal [12,14,20,44].

Both figures show that serotypes F, K and M are different from all other *O. rhinotracheale* strains and, while clustering into one branch, also quite different from each other.

It has been shown that MALDI-TOF MS database used for identification should include field strains to improve identification results [21,45]. Thus, the MSP’s of 47 field strains from this study were included in our in-house MALDI-TOF MS reference database and subsequently, all strains were re-identified. Certainly, MALDI identification scores improved for serotypes M, K and F but only to genus level (MALDI score 1.99, 1.81 and 1.79 respectively).

To further investigate the relationship between the strains belonging to serotypes F, K and M (LMG 18856, 18861, and 19032) sequencing on an Illumina MiSeq in 300bp paired-end mode was performed. Assembly of the genomes was done by using SPAdes and riboSeed [24,25]. The already fully sequenced *O. rhinotracheale* strains, DSM 15997 (GenBank: GCA_000265465.1), H06 030,791 (GenBank: GCA_000754515.1) and UMN 88 (GenBank: GCA_000756505.1) were used for comparison. While the resulting genomes were substantially more fragmented than the previously published *O. rhinotracheale* genomes, the gene content was similar, with all strains containing around 2300–2500 genes (Additional file S4. Annotation statistics). We also assessed the completeness of the genomes by looking for the conserved single-copy orthologues defined in the BUSCO (v3) *bacteroidetes_odb9* dataset in both the newly sequenced and the previously published genomes [27,28]. As a result, 438 of the 443 genes in all genomes were found, with slightly more fragmented ones in the newly sequenced genomes (8 vs. 6/7, Additional file S1. Assembly statistics).

Antibiotic treatment is an important intervention strategy in the case of *O. rhinotracheale* outbreaks [3]. In addition to the limitation of antibiotics which can be used in food-producing poultry, the increasing number of antibiotic multi-resistant strains, including resistance to tetracycline and macrolide, is recognized as a major problem worldwide [46,47,48,49,50]. Genes for antibiotic resistance were found in strains belonging to serotypes K (LMG 18861) and M (LMG 19032). These genes are responsible for macrolide and tetracycline resistance, namely erythromycin resistance methyltransferase (erm 23S ribosomal RNA methyltransferase (erm(35))) and tetracycline-resistant ribosomal protection protein (tetQ), respectively. Interestingly, no antibiotic resistance genes were found in the strain LMG 18,856 belonging to serotype F using the online and the command-line version of the RGI tool (v. 5.0) for the CARD database (v. 3.2) [23,24] (Additional file S2. Antibiotic Resistance CARD).

**Figure 3:**
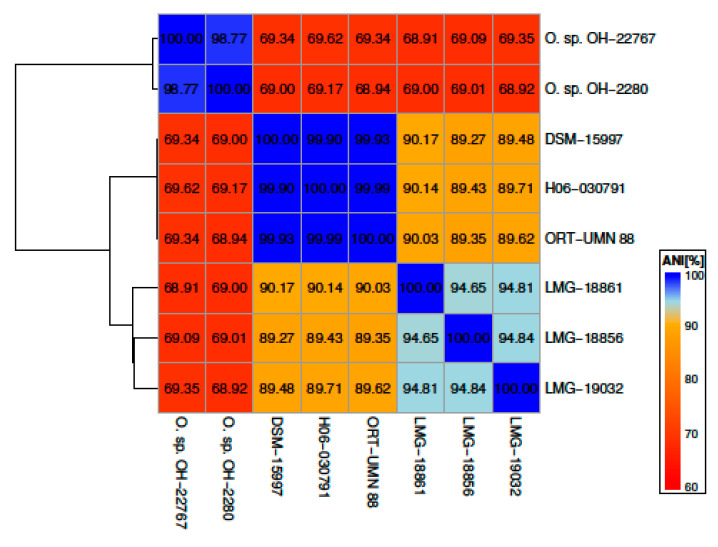
Heatmap of pairwise Average Nucleotide Identity (ANI) between the sequenced *O. rhinotracheale* and Cand. *O. hominis* (*O. sp. OH-22767 & OH-2280*) strains calculated using OrthoANI. ANI values are given in percent, the dendrogram on the right shows a simple hierarchical clustering on (1-ANI) using mean linkage.

We then calculated the overall average nucleotide identity (ANI) between all fully sequenced *O. rhinotracheale* strains and the two sequenced Cand. *O. hominis* strains (*O. sp. OH-22767* & *OH-2280*). The ANI of common sequences is closely correlated with the level of DNA-DNA hybridization (DDH), and a 95 to 96% ANI value is commonly used as a genomic measure of prokaryotic species delineation [32,51]. As expected, the two Cand. *O. hominis* strains show high sequence similarity (ANI: 98.7%) to each other but, are distant from the other strains (ANI ~69) (Figure 3). Unexpectedly, while the three previously sequenced *O. rhinotracheale* strains, DSM 15997, H06 030,791 and UMN 88, are highly similar to each other (ANI > 99%), they show only low similarity to the F, K and M serotype strains (ANI 89.3–90.2%), well below the usual species demarcation value of 95–96%. The F, K and M serotype strains are more similar to one another, but still slightly below 95% (ANI: 94.7–94.8%). Together, this indicates that these three newly sequenced strains belong to a different *Ornithobacter* species than *O. rhinotracheale*, they might even constitute multiple new species. In agreement with this assumption are reports from vaccination studies that showed cross-protection for the most frequently isolated serotypes but not for strains belonging to serotypes F, K and M. Until now it was concluded that these serotypes may be less virulent and play an inferior role in outbreaks as they are only occasionally isolated [17,52].

**Figure 4:**
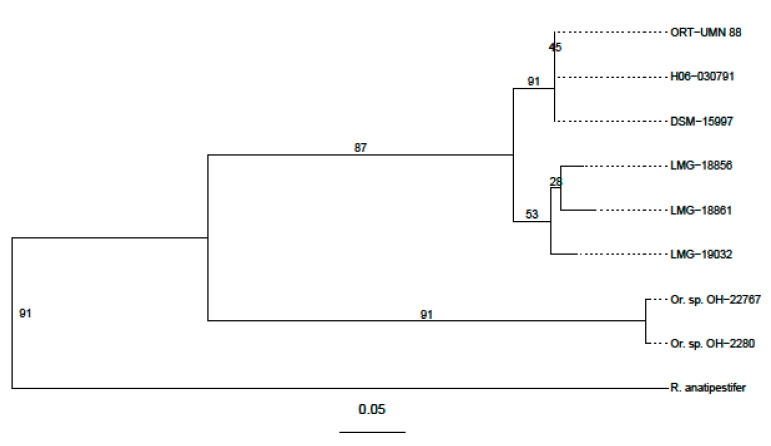
UBCG maximum likelihood multi-gene tree of the sequenced *O. rhinotracheale* and Cand. *O hominis* (*O. sp. OH-22767 & OH-2280*) strains using *Riemerella anatipestifer* (DSM 15868) as an outgroup. The tree was constructed RAxML with a general time-reversible model with gamma-distributed rate variation and 1000 bootstraps. The numbers display the gene support index (gsi), indicating how many of the gene trees support each split of the final concatenated tree. The gene encoding *tig* (Trigger factor) was not identified in any genome, so in the end, only 91 core genes were used.

To further assess the relationship between strains LMG 18856, 1886 and 19,032 to the other *Ornithobacter* strains and species, we used the UBCG (up-to-date bacterial core gene) tool [33]. With this, a phylogeny from a set of 92 predefined single-copy core genes from all fully sequenced *O. rhinotracheale* and Cand. *O. hominis* strains, as well as *Riemerella anatipestifer* (DSM 15868, Genbank: NC_017045.1) as an out-group, was constructed. UBCG constructs a tree from a concatenation of all genes, as well as single-gene trees, and gives the number of gene trees supporting each branch in the concatenated tree as a gene support index (gsi), as a measure of robustness. As one gene-*tig*-was not identified in any of the genomes, the maximal gsi for our study was 91. The concatenated UBCG tree shows a clear separation between the *O. rhinotracheale* and Cand. *O. hominis* strains (gsi: 91), as well as a distinct separation into two clades between the previously sequenced and the actual LMG strains (gsi: 87) (Figure 4).

**Figure 5:**
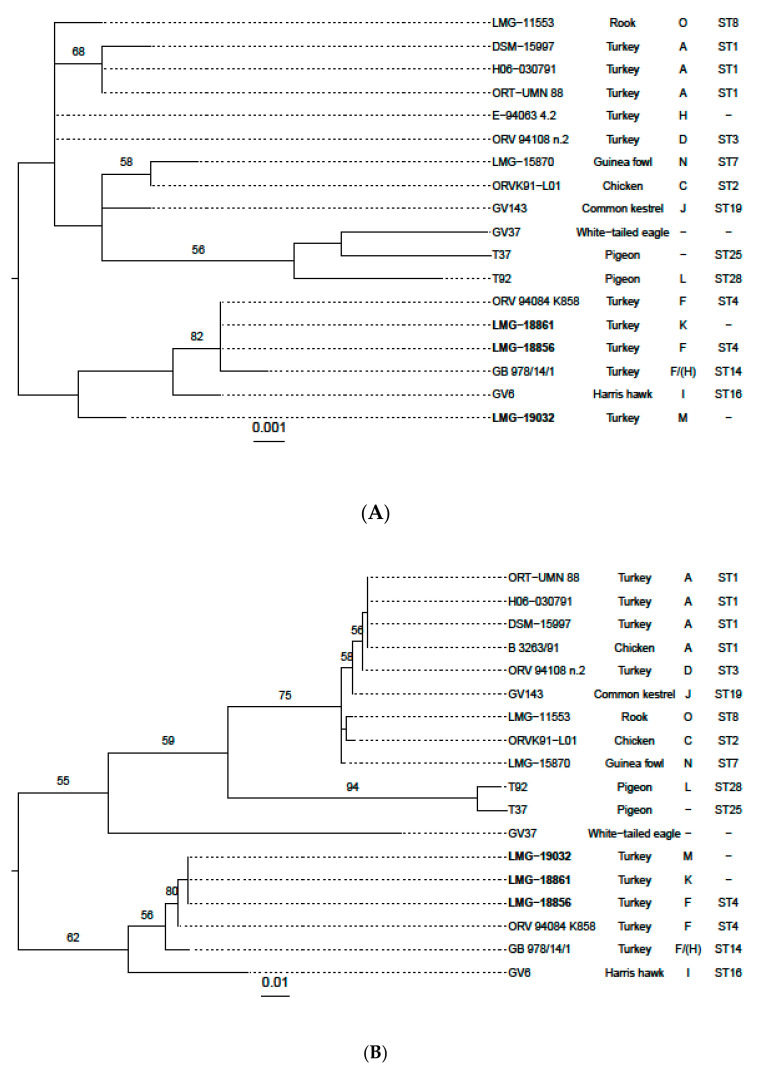
Maximum likelihood tree constructed from 16S-RNA (**A**) and rpoB (**B**) sequences using RAxML with a general time-reversible model with gamma-distributed rate variation and 1000 bootstraps. Bootstrap values greater than 50 percent are shown on branches. Sequences from Cand. *O. hominis* (*O. sp. OH-22767 & OH-2280*) were used as an out-group for rooting. The text columns give host, serotype and sequence type (ST) according to PubMLST. The newly sequenced strains are indicated in boldface. For strains with identical sequences only one was included for calculations (16S-RNA: ORV 94,084 K858, LMG-18861, LMG-18856; ORT-UMN88, H06−030791; E−94063 4.2, ORV 94,108 n.2, rpoB: ORT−UMN 88, H06−030791, DSM−15997, B 3263/91; LMG−18856, LMG−18861, LMG−19032).

We also wanted to explore, how our newly sequenced strains harmonize with others, not fully sequenced *O. rhinotracheale* strains. For this, we compared the sequences of the 16S rRNA and the beta subunit of the RNA polymerase (*rpoB*) extracted from the genomic sequences with published sequences from various reference and field strains [10,13]. The two Cand. *O. hominis* strains were used as an outgroup (Additional file S5. List of Sequences used). The resulting trees (Figure 5A,B) show a split into two clades from the outgroup. One clade contains six isolates comprising the newly sequenced M, F and K serotype strains, as well as some previously isolated strains of which two belong to serotype F and one to serotype I. The other clade includes 12 previously sequenced other strains, most of them belonging to serotype A.

**Figure 6:**
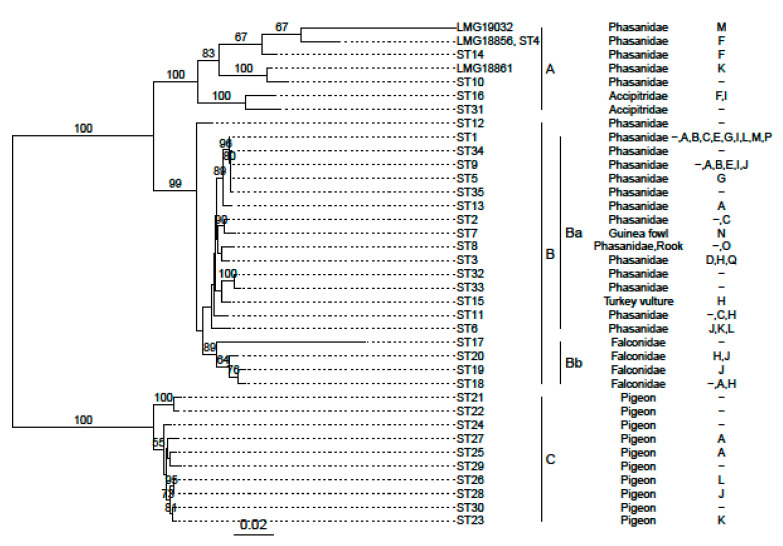
Maximum likelihood tree constructed from the concatenated sequences of the genes used for MLST using RAxML with a general time-reversible model with gamma-distributed rate variation and 1000 bootstraps. Bootstrap values greater than 50 percent are shown on branches. At the tips, either the sequence type (ST) or the strain name is given, followed by the host and the determined serotypes (slight serotype cross-reactions are excluded, for the full list see Additional file S5. Pubmlst). The lines indicate the clusters and sub-clusters described in [10].

As the 16S rRNAs show little divergence, and only a subset of known strains have *rpoB* sequences available, we also compared our newly sequenced strains to multilocus sequence typing (MLST) data from PubMLST [10,39]. While LMG 18,856 (serotype F) can be typed as ST4, the other two LMG strains constitute new MLSTs (Additional file S6. PubMLST typing). We created a phylogenetic tree from a concatenation of the sequences used for multilocus sequence typing to see where our newly sequenced strains would fit (Figure 6). They all fall into the previously described cluster A [10], which only contains strains of serotypes M, F, K and I together with 2 strains not assigned to a specific serotype.

## 4. Conclusions

In the present study, we demonstrate that MALDI-TOF MS can be used for the identification and characterization of *O. rhinotracheale*. By including a high number of field strains in the in-house MALDI-TOF MS reference database the identification level could be substantially improved. MALDI-TOF MS confirmed previous findings regarding the regional clonality of *O. rhinotracheale* strains which seem to stay stable over years. Although the distinction of serotypes was not possible with MALDI-TOF MS we were able to show that strains belonging to serotypes F, M and K do not belong to the species *O. rhinotracheale*. This finding was confirmed by whole genome sequencing data indicating that these three serotypes are clearly different to the other *O. rhinotracheale* strains, and probably belong to different *Ornithobacter* species.

## Figures and Tables

**Table 1:** Established MALDI-TOF MS Reference database.

No.	Strain	Remark	Year of Isolation	Geographic Origin	Source	MALDI Score
1	LMG 09085	Serotype A	1988	United Kingdom	Turkey	2.54
2	LMG 09086	Type strain	1988	United Kingdom	Turkey	2.51
3	LMG 09087		1988	United Kingdom	Turkey	2.47
4	LMG 09088	Serotype A	1988	France	Turkey	2.50
5	LMG 10958		1991	Belgium	Turkey	2.37
6	LMG 10960		1988	Belgium	Turkey	2.54
7	LMG 10961		1989	Belgium	Turkey	2.46
8	LMG 10967		1990	Belgium	Chicken	2.45
9	LMG 10968		1990	Belgium	Chicken	2.49
10	LMG 10969		1990	Belgium	Chicken	2.45
11	LMG 11342		1991	Belgium	Partridge	2.21
12	LMG 11343		1989	Belgium	Chicken	2.11
13	LMG 11553	Serotype O	1983	Germany	Rook	2.40
14	LMG 11554	Serotype H	1983	Germany	Rook	2.28
15	LMG 11555	Serotype H	1983	Germany	Rook	2.26
16	LMG 12589		1991	Germany	Turkey	2.41
17	LMG 12590		1992	South Africa	Broiler	2.49
18	LMG 12591		1992	South Africa	Broiler	2.36
19	LMG 12599		1991	Germany	Turkey	2.55
20	LMG 12600		1992	South Africa	Broiler	2.49
21	LMG 13111		1992	Belgium	Poultry	2.47
22	LMG 13114	Serotype N	1992	Belgium	Guinea Fowl	2.27
23	LMG 14529		1993	The Netherlands	Turkey	2.49
24	LMG 14530		1993	The Netherlands	Turkey	2.53
25	LMG 14578		1994	The Netherlands	Turkey	2.41
26	LMG 15497		1992	Israel	Turkey	2.45
27	LMG 15501		1993	Israel	Turkey	2.43
28	LMG 15502		1993	Israel	Turkey	2.43
29	LMG 15503		1993	Israel	Turkey	2.44
30	LMG 15504		1993	Israel	Turkey	2.43
31	LMG 15505		1993	Israel	Turkey	2.47
32	LMG 15506		1993	Israel	Turkey	2.48
33	LMG 15507		1993	Israel	Turkey	2.28
34	LMG 15508		1993	Israel	Turkey	2.45
35	LMG 15509		1993	Israel	Turkey	2.41
36	LMG 15510		1993	Israel	Turkey	2.38
37	LMG 15511		1993	Israel	Turkey	2.51
38	LMG 15513		1993	Israel	Turkey	2.35
39	LMG 15517		1993	Israel	Turkey	2.44
40	LMG 15520		1993	Israel	Turkey	2.40
41	LMG 15521		1994	Israel	Turkey	2.38
42	LMG 15523		1994	Israel	Turkey	2.37
43	LMG 15524		1994	Israel	Turkey	2.36
44	LMG 15525		1994	Israel	Turkey	2.37
45	LMG 15870		1995	Belgium	Guinea Fowl	2.23
46	LMG 15871		1995	Belgium	Turkey	2.58
47	LMG 18851	Serotype A^ref^	1991	South Africa	Chicken	2.42
48	LMG 18852	Serotype B^ref^	1991	Germany	Turkey	2.45
49	LMG 18853	Serotype C^ref^	1991	United States	Chicken	2.24
50	LMG 18854	Serotype D^ref^	1994	France	Turkey	2.31
51	LMG 18855	Serotype E^ref^	1995	France	Chicken	2.40
52	LMG 18856	Serotype F^ref^	1994	South Africa	Turkey	1.54
53	LMG 18857	Serotype G^ref^	1994	South Africa	Chicken	2.46
54	LMG 18858	Serotype H^ref^	1994	Netherlands	Turkey	2.38
55	LMG 18859	Serotype I^ref^	1998	United States	Turkey	2.50
56	LMG 18860	Serotype J^ref^	1998	The Netherlands	Turkey	2.40
57	LMG 18861	Serotype K^ref^	1997	United States	Chicken	1.69
58	LMG 18862	Serotype L^ref^	1997	United Kingdom	Turkey	2.30
59	LMG 19032	Serotype M^ref^	1998	France	Turkey	1.90

ref = reference strain of serotype.

**Table 2:** *Ornithobacterium rhinotracheale* field strains isolated from Austrian turkey flocks.

Farm	Strain Name	Area	MALDI Score Value
1	02/0103 *	Lower Austria	2.40
	05/0091-1		2.50
	16/01621		2.48
2	02/0301	Lower Austria	2.53
	03/0638-3		2.46
3	02/0658	Lower Austria	2.54
4	03/0525	Lower Austria	2.53
	03/0526-3		2.58
5	03/0530	Upper Austria	2.59
6	03/2652	Lower Austria	2.58
7	03/3567	Upper Austria	2.54
8	04/0372	Burgenland	2.49
	04/0372-3		2.53
9	04/0471-1	Lower Austria	2.61
	04/4559-1		2.54
	04/4559-2		2.53
10	04/1260	Upper Austria	2.53
	06/07788		2.61
11	04/1972-2	Lower Austria	2.53
12	04/3032	Upper Austria	2.54
13	04/3086	Lower Austria	2.54
14	04/4018	Upper Austria	2.51
15	04/4426-1	Lower Austria	2.50
16	04/4519	Lower Austria	2.63
17	04/4519-2	Lower Austria	2.51
18	06/05549	Upper Austria	2.54
	13/00573		2.38
19	06/8251-2	Burgenland	2.48
	06/8251-3		2.38
	06/8251-5		2.46
20	08/07315-2	Burgenland	2.72
21	08/07581	Lower Austria	2.61
	15/25053		2.51
22	08/09353	Lower Austria	2.63
23	08/18891	Lower Austria	2.54
24	10/11996	Lower Austria	2.46
	10/11997		2.51
	16/22784		2.46
25	13/00771	Burgenland	2.56
26	13/01156	Lower Austria	2.47
27	13/04346	Upper Austria	2.52
	14/02621		2.46
	16/22481		2.39
28	13/22716	Lower Austria	2.50
29	14/00974	Upper Austria	2.50
30	15/19094	Lower Austria	2.43
31	16/00975	Lower Austria	2.70

* year of isolation/internal diagnostic number.

## Data Availability

The sequence data are available at GenBank under the Bioproject PRJNA501809 with sample IDs SAMN10346233, SAMN10346254, and SAMN10346265. The assemblies are available at the NCBI under the GenBank IDs GCA_009659665.1, GCA_009659645.1, and GCA_009659705.1.

## Supplementary Materials

The following are available online at https://www.mdpi.com/article/10.3390/microorganisms9051006/s1. Additional file S1. Assembly statistics: Metrics and statistics for the assemblies created in this study (strains LMG 18856, 18861 and 19032) and of previously sequenced *O. rhinotracheale* strains (DSM 15997, ORT-UMN 88 and H06_030791) for comparison. L50 and L90 give the smallest number of contigs of the assembly whose summed length is greater than 50 or 90% of the total assembly length. N50 and N90 give the minimal length of these contigs. Under BUSCO the number of single-copy core genes (out of 443 total genes) found complete, duplicated, fragmented or missing in the assembled genomes are given. Additional file S2. Antibiotic Resistance CARD: Antibiotic resistance genes using the online and the command-line version of the RGI tool (v5.0) for the CARD database (v3.2). Additional file S3. MLST typing: Sequence types obtained computationally for the LMG strains using stringMLST and the *O. rhinotracheale* MLST schemes from PubMLST. Additional file S4. Annotation statistics: Breakdown of the major results of the NCBI Prokaryotic Genome Annotation Pipeline for the newly and previously sequenced *O. rhinotracheale* strains. Different versions of the pipeline were used for previously sequenced strains. Additional file S5. Sequences used: List of sequences used for this article. Additional file S6. pubMLST: Table of *O. rhinotracheale* strains, hosts, origins, serotypes and sequence types obtained from PubMLST on the 01/24/2020. Serotypes in brackets indicate slight cross-reactions.
